# Vibrational and Resistance Responses for Ether-Amine Solutions of the Buckypaper-Based Chemiresistor Sensor

**DOI:** 10.3390/nano15151197

**Published:** 2025-08-05

**Authors:** Débora Ely Medeiros Ferreira, Paula Fabíola Pantoja Pinheiro, Luiza Marilac Pantoja Ferreira, Leandro José Sena Santos, Rosa Elvira Correa Pabón, Marcos Allan Leite Reis

**Affiliations:** 1 Postgraduate Program in Materials Science and Engineering, Federal University of Para, Ananindeua 67130-660, PA, Brazil; debora.ferreira@ananindeua.ufpa.br (D.E.M.F.); leandro.santos@abaetetuba.ufpa.br (L.J.S.S.); 2 3D Nanostructuring Laboratory, Federal University of Para, Belem 66075-110, PA, Brazil; paulapinheiro@ufpa.br (P.F.P.P.); luiza.ferreira@cameta.ufpa.br (L.M.P.F.); 3 Vale Technological Institute, Ouro Preto 35400-000, MG, Brazil; rosa.correa@itv.org; 4 Postgraduate Program in Amazon Natural Resources Engineering, Federal University of Para, Belem 66075-110, PA, Brazil; 5 Postgraduate Program in Geology and Geochemistry, Federal University of Para, Belem 66075-110, PA, Brazil

**Keywords:** buckypaper, sensor, ether-amine, selectivity

## Abstract

The development of miniaturized sensors has become relevant for the detection of chemical/biological substances, since they use and detect low concentrations, such as flocculants based on amines for the mining industry. In this study, buckypaper (BP) films based on carboxylic acid functionalized multi-walled carbon nanotubes (f-MWCNTs) were produced through vacuum filtration on cellulose filter paper to carry out sensory function in samples containing ether-amine (volumes: 1%, 5%, 10% and 100%). The morphological characterization of the BPs by scanning electron microscopy showed f-MWCNT aggregates randomly distributed on the cellulose fibers. Vibrational analysis by Raman spectroscopy indicated bands and sub-bands referring to f-MWCNTs and vibrational modes corresponding to chemical bonds present in the ether-amine (EA). The electrical responses of the BP to the variation in analyte concentration showed that the sensor differentiates deionized water from ether-amine, as well as the various concentrations present in the different analytes, exhibiting response time of 3.62 ± 0.99 min for the analyte containing 5 vol.% EA and recovery time of 21.16 ± 2.35 min for the analyte containing 10 vol.% EA, revealing its potential as a real-time response chemiresistive sensor.

## 1. Introduction

In the reverse cationic flotation process of iron ore, ether-amines are the cationic collectors that allow quartz to float and isolate it from iron oxide. The collectors have a molecular structure composed of a covalent part (nonpolar and hydrophobic hydrocarbon chain) and an ionic part (which is an ether-amine with a permanent dipole), that are both amphipathic. Primary amines (RNH_2_) are highly insoluble, but the transition to primary ether-amine R-O-(CH_2_)_3_-NH_2_ gives it greater solubility due to the existence of the C-O covalent bond, which allows it to reach the solid–liquid and liquid–gas interfaces. Additionally, moderate neutralization of the ether-amine, often done with acetic acid, further benefits its solubilization [1,2].

Due to its high market value and high consumption, as the ore becomes scarcer, ether-amine becomes a constant source of concern, as it entails high operating costs and is an essential element in the beneficiation process. According to Batisteli and Peres [3], an iron ore processing company uses approximately 1500 tons of amine per year to produce approximately 16 million tons of concentrate per year, representing approximately 50% of the total reagent costs. Research conducted by Silva [4] revealed that the liquid float discharged from tailings dams contains 45% of the applied ether-amine, which varies in concentration between 5 and 45 mg·L^−1^. Globally, the ether-amine market is estimated to reach approximately USD 1.6 billion by the end of 2025, driven by the growth of iron mining in Latin America. Therefore, real-time detection of this reagent will facilitate the reuse of ether-amine and, consequently, reduce the overall reagent costs. In addition to economic waste, environmental contamination can reduce the self-purification capacity of rivers by decreasing oxygen absorption. It can also harm human health, as most amines cause irritation to the skin, eyes, mucous membranes, and respiratory tract [2,5].

Therefore, mining industries are becoming more stringent regarding the reuse of ether-amine contained in flotation tailings. To meet market demands, the development of low-cost, easy-to-operate, automatic analytical systems that enable accelerated and accurate detection of ether-amine concentrations in tailings is highly relevant, as these methods can help reduce industrial costs and environmental pollution. Currently, techniques for detecting ether-amines in ore flotation wastewater, such as chromatography, colorimetry, and Fourier transform infrared (FTIR) spectroscopy [6,7], require complex and expensive processes and equipment.

A little-explored alternative for this application that can simplify detection and reduce operating costs is the use of a device capable of providing an analytical signal about changes in the chemical composition of the surrounding environment, such as chemiresistive sensors [8]. Their simple configuration provides low cost and portability, as well as selectivity (distinguishing different chemical substances), reproducibility, recovery (allowing reuse), and a significant output signal for any quantity of the target element in real time [9]. The molecule of interest for detection is called the analyte. First, the analyte interacts with the sensitive element exposed on the detector, which has some electronic compatibility with it. The sensitive element (or active layer) of the device can be made up of polymeric materials, nanoparticles, or molecular elements called receptors. After adsorption of the analyte on the active layer, some particularities of the sensor may change, such as electrical resistance, which will be recognized and transformed into a compatible signal by the transduction assignment [10,11].

The production of miniaturized chemiresistor sensors can add more advantages, as it tends to maximize their performance by integrating electrodes with chemically altered faces, starting with the introduction of strongly sensitive catalysts such as CNTs [12]. CNTs are relevant to a wide range of applications due to their one-dimensional (1D) structure, which gives them good mechanical, thermal, and electrical characteristics, as well as the low density and large length/diameter aspect ratio that determines their large unit surface area. CNTs were first elucidated by the microscopist Sumio Iijima in 1991 [13] and, since then, they have been of great importance due to their diverse electronic capabilities.

Geometrically, when they are organized only by a sheet of extruded graphene giving rise to hollow cylinders, they are classified as single-walled carbon nanotubes (SWCNT); if they are organized by two concentric cylinders, they are classified as double-walled carbon nanotubes (DWCNT); when they are made up of several concentric cylinders, they are identified as multi-walled carbon nanotubes (MWCNTs) [14,15]. However, the practical application of CNTs presents challenges in the field of nanotechnology due to their small size scale. A current concept for resolving this impasse, with a view to characterizing their particularities, involves the development of a macroscopic porous sheet made up of a network of CNTs, called buckypaper (BP) [16].

BPs are films formed by a macroscopic arrangement of entangled CNTs or randomly dispersed nanocomposites, structured as a planar film on a filtration membrane (e.g., paper). This membrane can also be removed after the film is produced, resulting in a self-supporting film. Furthermore, they exhibit flexibility, lightness, porosity, and easy to handle. The entangled CNTs are held together by Van der Waals interactions at the tube-tube junctions and can have high mechanical strength, excellent electrical conductivity, sensitivity, and chemical selectivity [17,18]. Ferreira et al. [18], for example, developed a chemiresistive sensor using BP based on carboxylic acid (COOH) functionalized MWCNTs (f-MWCNTs) for Port wine analysis, and the reported time-dependent response results showed that the BP sensor was able to distinguish unadulterated wines from those adulterated with water and alcohol, emitting a distinct response signal depending on the type of adulterating agent added.

Therefore, within this context, this study aims to evaluate the potential of BP based on f-CNTs/cellulose as a sensor element for real-time detecting residual ether-amine contained in different process water flow rates, with the aim of reusing/repurposing it and contributing to cost reduction in the iron ore flotation process. For this purpose, a BP film was developed from f-MWCNTs to perform the function of an ether-amine (EA) sensor at 100 vol.% or intentionally modified with deionized water at concentrations of 1 vol%, 5 vol%, or 10 vol%. The response of the sensor element to the different concentrations of analyte and deionized water was researched to consider its ability to differentiate the types of solutions and their varying volumes of ether-amine.

## 2. Materials and Methods

### 2.1. BP Production

**Figure 1:**
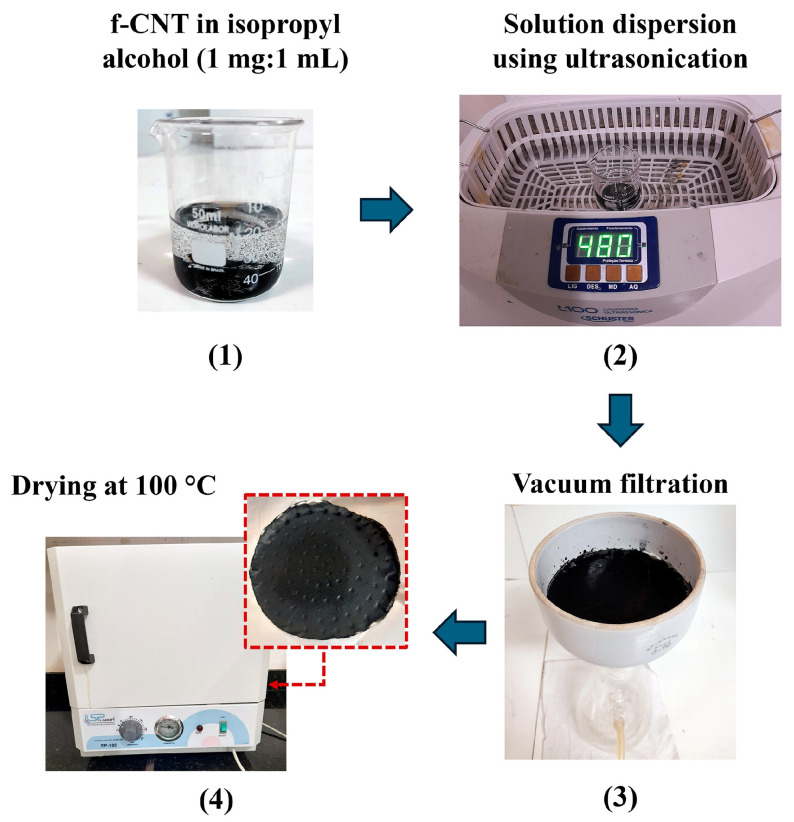
Schematic representation of the buckypaper production stages.

Functionalized carbon nanotubes (f-CNTs) were purchased from Nanoview^®^ Nanotecnologia (Belo Horizonte, Brazil), with 95% purity (external diameter of 10–30 nm and length of 1–10 μm). The nanotubes were (1) dispersed in isopropyl alcohol in a 1:1 ratio and kept under agitation for (2) 1 h using an ultrasound (SCHUSTER, Santa Maria, Brazil, model L100). Afterward, the solution was (3) vacuum filtered through qualitative filter paper (grammage: 80 g/m^2^; nominal thickness: 205 μm; porosity: 14 μm, manufactured by J PROLAB, São José dos Pinhais, Brazil). The paper was placed in a Buchner funnel (diameter: 12 cm; CHIAR-ROT, Maua, Brazil) attached to a Kitasato (500 mL; SPLABOR, Presidente Prudente, Brazil) connected to the vacuum pump (nominal vacuum of 2 × 10^−2^ mmHg and power: 1/3 HP; SYMBOL VACUUM TECHNOLOGY LTDA, Sumaré, Brazil). Finally, (4) the BP based on f-CNTs/cellulose was taken to an oven at 100 °C to dry the solvent for 1 h. This BP production method follows the steps and procedures described by Pinheiro et al. [19]. Figure 1 shows all the steps of BP production.

### 2.2. Morphological Characterization of BPs

The morphological characterization of the BP produced was carried out by scanning electron microscopy (SEM). For this purpose, a VEGA3 microscope (TESCAN, Brno, Czech Republic) was used in secondary electron (SE) detection mode to analyze the top and cross sections of the sample, with a voltage of 20 kV and 5 kV and working distances of 6.01 mm and 24.22 mm, respectively. To analyze the cross section of the BP, the sample was fractured after immersion in liquid nitrogen.

### 2.3. BP Sensor Chemiresistive Test

**Figure 2:**
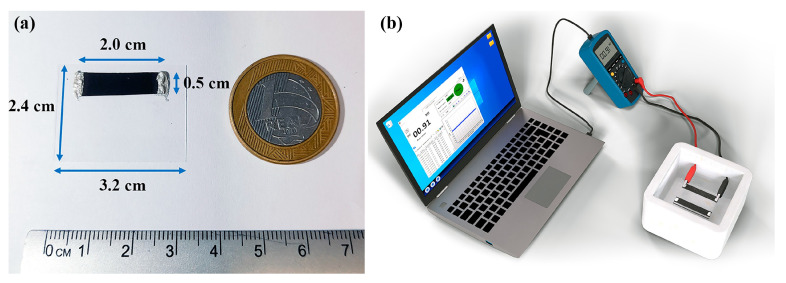
Schematic illustration showing (**a**) sensor element made with the dimensions of the BP and the coverslip and (**b**) setup for chemiresistive characterization of the sensor element.

To make the sensor element, 14 BP samples were taken from the regions between the center and the edge of the film, measuring 2.0 cm in length and 0.5 cm in width, as shown in Figure 2a. Later, they were fixed to glass coverslips (2.4 cm × 3.2 cm) using commercial silver conductive ink (NANOVIEW^®^ NANOTECNOLOGIA, Belo Horizonte, Brazil). The chemiresistive test was followed by monitoring electrical resistance as a function of time (R × t), by two-point measurement, using a digital multimeter ET-2232 (0.1 Ω–0.01 MΩ resolution, accuracy ±1.2–1.5% Reading + 2Digits, MINIPA, Joinville, Brazil) connected to the notebook by USB, as shown in Figure 2b.

The sensing element was tested in the presence of deionized water, ether-amine 100 vol.% (commercial Flotigam^TM^ EDA, produced by CLARIANT, acquired from the Vale Institute of Technology), and a mixture of 1 vol.% of ether-amine (0.02 mL) diluted in deionized water (1.98 mL), as well as the concentrations of 5 vol.% of ether-amine (0.1 mL) in deionized water (1.9 mL) and 10 vol.% of ether-amine (0.2 mL) in deionized water (1.8 mL). The pH of all the analytes was previously measured with a YY-1030 pH meter (YIERYI, Shenzhen, China), in view of this 10 mL samples of deionized water (DW), 1 vol.% ether-amine (EA1), 5 vol.% ether-amine (EA5), 10 vol.% ether-amine (10 EA) and 100 vol.% ether-amine (EA100) were tested at room temperature (25 °C), resulting in pH 7.00, pH 7.40, pH 7.50, pH 7.60, and pH 8.40, respectively.

To assess the distinguishability, reproducibility, and recovery of the BP response during three measurement cycles (except for EA100), 5 μL of each analyte (DW, EA1, EA5, EA10 or EA100) was added to the surface of the sensor element samples (at room temperature) using a monochannel micropipette graduated in microliters (variable volume: 0.1–10 μL; HTL AS, Warsaw, Poland) by dripping analogous to the process used by Yakhno et al. [20]. The response of the sensing element to the chemical stimulus was calculated by Equation (1) based on the change in normalized relative electrical resistance. Where $R_{f}$ is the final electrical resistance of the sensor element after application of the analyte and $R_{0}$ is the initial resistance without analyte, both measured at room temperature.

(1) $$\mathrm{Response}\;(\%)=\left[\left(R_{f}-R_{0}\right)/R_{0}\right]\;\times \;100$$

### 2.4. Multivariate Statistical Analysis

The maximum response, response, and recovery time data collected from the tests with analytes EA1, EA5, and EA10 were treated by the principal component analysis (PCA) technique, using the free statistical software PAST (version 4.09) developed by Hammer et al. [21]. Initially, the data was pre-processed to reduce the influence of variables with greater weight on the main components generated, due to their distinct units, as done in [18] using the relationship given in Equation (2).

(2) $$z=\left(X-\bar{X}\right)/s$$

where z is the z-score resulting from the linear transformation, X is the raw score obtained in each measurement, $\bar{X}$ is the arithmetic mean of the raw scores, and s is the standard deviation of the sampling distribution. In processing, the average maximum response, response time and recovery data (obtained from the 3 cycles for each analyte) were used as input variables composing an original data matrix of type *n* × *p* (where *n* corresponds to the samples (lines) and *p* to the input variables (columns), as described by Kherif and Latypova [22]. In linear transformation, a new reduced group of variables called principal components (PCs) is generated by the linear combination of the original data matrix, through a decomposition process where there is no significant loss of chemical information [23].

### 2.5. Vibrational Characterization of the BP Sensor with Analytes

The vibrational characterization of the f-CNTs as received, the BP as produced (i.e., without analyte) and the BPs with the different analytes (i.e., BP + DW, BP + EA1, BP + EA5, BP + EA10 and BP + EA100) was carried by Raman spectroscopy, using a HORIBA LabRAM HR Evolution spectrometer (spectral resolution around 0.3–0.4 cm^−1^, HORIBA, San Francisco, CA, USA) at room temperature, a laser line with a wavelength of 633 nm, a 20× objective lens, an acquisition time of 30 s in 2 accumulations, a power filter calibrated at 100%, laser power of 0.39 mW and a range of 40–4000 cm^−1^. Each BP sample was exposed to a single drop of 5 μL of one of the analytes and then, after three months, they were subjected to vibrational analysis.

## 3. Results

### 3.1. BP Morphology

**Figure 3:**
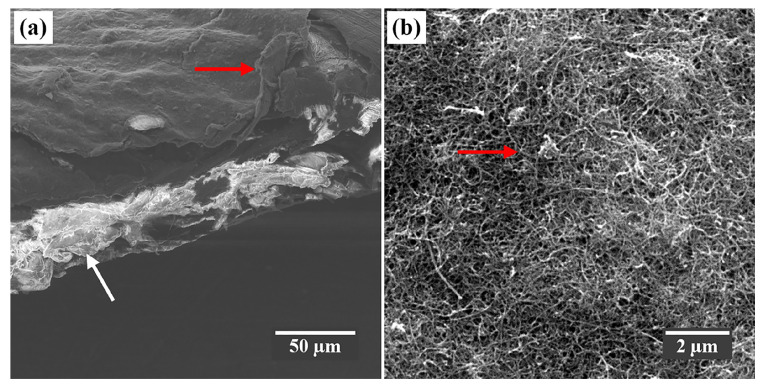
SEM micrograph of the BP in (**a**) cross and (**b**) superior view, at 894× and 17,700× magnifications, respectively.

The morphological characterization of BP by scanning electron microscopy showed a layer of f-CNTs (red arrow) dispersed over the surface of the paper’s cellulose fibers (white arrow) and impregnated among its pores, as shown in the micrograph in Figure 3a. Figure 3b shows that this layer is composed of randomly oriented f-CNT agglomerates (red arrow).

### 3.2. Electrical Responses

**Figure 4:**
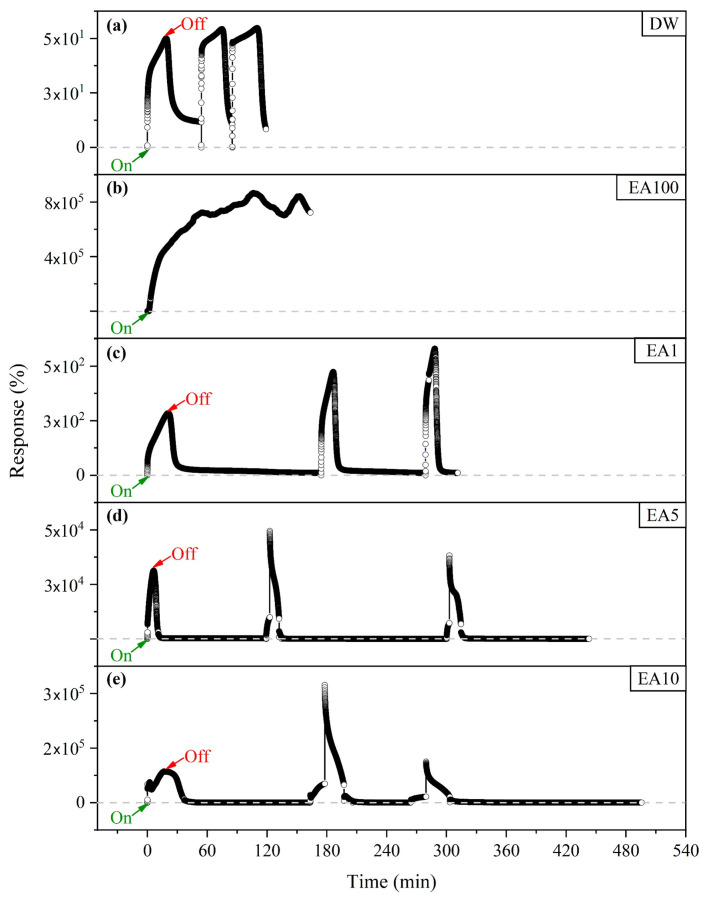
Comparative Response (%) as a function of time for 5 µL of (**a**) deionized water (DW), (**b**) ether-amine 100 vol.% (EA100), and at concentrations of (**c**) 1 vol.% (EA1), (**d**) 5 vol.% (EA5), and (**e**) 10 vol.% (EA10). In On, the analyte is dripped, and in Off, it starts sensor recovery.

Figure 4 shows the comparative Response (%) curves as a function of time obtained for the sensor element samples in the presence of 5 μL of: deionized water (DW), 100 vol.% ether-amine (EA100), and at concentrations of 1.0 vol.% (EA1), 5.0 vol.% (EA5), and 10 vol.% (EA10). The results show that the BP response for EA100 can reach 723.974% (in a single drip), and at different concentrations, it also differs from the response to DW (52.99 ± 2.64%). The BP response for the EA1, EA5 and EA10 concentrations (pH 7.5 ± 0.1) were 446.25 ± 150.14%, 39,699.14 ± 9154.10% and 174,241.57 ± 130,005.32%, respectively.

The response and recovery times (together referred to as the reaction time) were extracted from the response curves in Figure 4 and are shown in Table 1. They are determined as the intervals essential for the sensor to reach 90% response to the analyte and return 90% of the original baseline signal, respectively [24]. The response time for the different concentrations of analytes changed, being 43.57 min for 100 vol.% EA, 11.49 ± 4.97 min for 1 vol.% EA, 3.62 ± 0.99 min for 5 vol.% EA, and 14.86 ± 1.32 min for 10 vol.% EA. Similarly, the recovery time (which serves as a parameter to evaluate the possibility of reusing the BP sensor) for 10 vol.% EA was approximately 2.5 to 2.3 times longer than for 5 vol.% EA and 1 vol.% EA, respectively.

Thus, it is noted that the higher the concentration of ether-amine, the greater the electrical response of the BP, whose sensitivity is characterized by an increase in the variation of the normalized relative resistance for low quantities of the analytes. Furthermore, in three measurement cycles, the electrical resistance of the BP returned to the initial value as the solution evaporated, demonstrating reproducible and recovery behavior.

### 3.3. Principal Component Analysis

**Figure 5:**
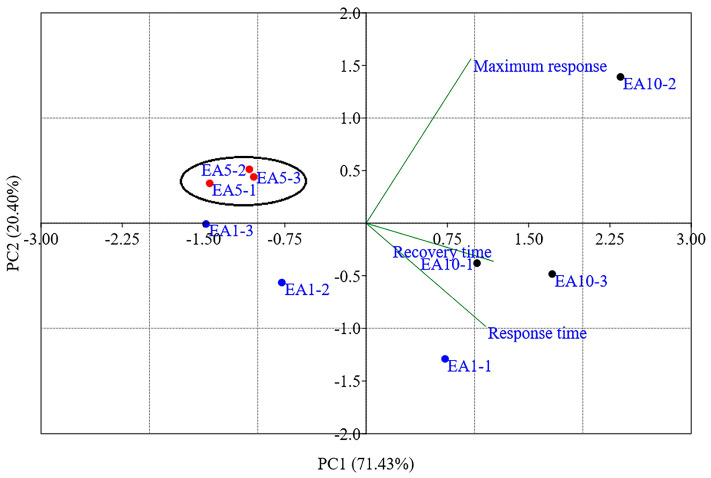
PCA Biplot generated with two PCs (PC1: 71.43% and PC2: 20.40%) for 3 cycles of samples EA1, EA5, and EA10.

The result of data processing using PCA is shown in the graph in Figure 5. During processing with the software, three principal components (PCs) were extracted: PC1, PC2, and PC3. However, the PCA graph was generated via the covariance matrix, using the first two PCs (PC1: 71.43% and PC2: 20.40%), as both correspond to 91.84% of the total variance of the data, a value greater than 80%, which makes it possible to represent the data set by the first two PCs [22,25]. The eigenvalue data and the variance explained by each PC are listed in Table S1 of Supplementary Materials.

The PCA Biplot presents scores corresponding to the samples of each analyte investigated in the four quadrants and loading vectors representing the analyzed input variables (maximum response, response time, and recovery time). The upper right quadrant shows the score for cycle 2 of sample EA10, while cycles 1 and 3 are shown in the lower right quadrant, in addition to cycle 1 of sample EA1. The upper left quadrant contains a cluster of cycles from sample EA5 (highlighted by the black circle) and scores from cycles 2 and 3 of sample EA1. The arrangement of the sample scores in distinct quadrants demonstrates the sensor’s ability to detect and distinguish samples with concentrations of 1 vol.%, 5 vol.%, and 10 vol.% of commercial ether-amine.

### 3.4. Vibrational Effects of Interactions Between CNTs and Analyte

**Figure 6:**
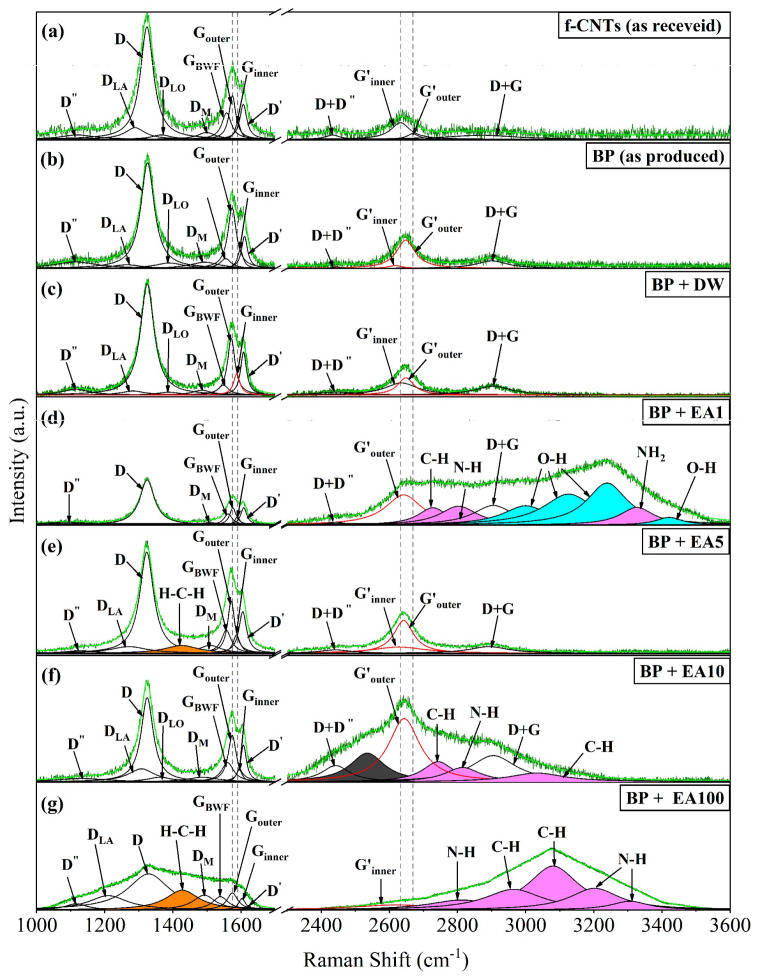
Raman spectra showing the bands and sub-bands obtained by Lorentzian deconvolutions (**a**) f-CNTs (as received), (**b**) BP without analyte (as produced), (**c**) BP + DW, (**d**) BP + EA1, (**e**) BP + EA5, (**f**) BP + EA5 and (**g**) BP + EA100.

The Raman spectra adjusted with Lorentzian deconvolutions, as reported in the works by Rebelo et al. [26], Reis et al. [27] and Santos et al. [28], for the samples of f-CNTs (as received), BP (as produced) and BPs after analyte dripping (BP + DW, BP + EA1, BP + EA5, BP + EA10 and BP + EA100) are compared in Figure 6a–g. In all the spectra, the characteristic vibrational signature of CNTs can be seen, designated by the presence of the D and G bands in the 1100–1450 cm^−1^ and 1525–1700 cm^−1^ regions, respectively [29].

Around the D band are shown sub-bands or “satellite bands” that occur due to the structural changes generated on the surface of the MWCNTs as a result of chemical functionalization and solubilization processes, labeled as: D” (associated with double resonance of longitudinal acoustic phonons with defects), D_LA_ and D_LO_ (referring to the longitudinal acoustic and optical phonon components, respectively), and D_M_ (whose subscript ‘M’ refers to Middle, because it is located between the D and G bands, around 1450–1500 cm^−1^ and, corresponds to the vibrational signature of amorphous carbon) [28,30].

The G-band region was decomposed into three sub-bands: G_outer_ (at 1571–1576 cm^−1^) and Ginner (at 1586–1603 cm^−1^), associated with the outermost and innermost tubes, respectively, and D’ (like a shoulder in the G band, defect-activated, at 1604–1623 cm^−1^). The G’ band (derived from a double electron-phonon resonance mechanism), in the 2500–3000 cm^−1^ region, is composed of the ${G^{\prime }}_{inner}$ and ${G^{\prime }}_{outer}$ sub-bands, also related to the innermost and outermost diameter distributions of the MWCNTs, respectively. Furthermore, the D + D” band derived from the combination/overtones of the phonons of the D and D” bands and the D + G band originating from the combination of the vibrational modes of the D and G bands through high-frequency double resonance processes are also observed [27,28,31].

Figure 6d shows the Raman spectrum of the analyte composed of the dispersion of ether-amine (1 vol.%) in Deionized Water (99 vol.%). The bands present in the 3000–3238 cm^−1^ and 3420 cm^−1^ regions (the areas of which are highlighted in cyan) may correspond to the stretching vibrations of the O-H bonds in water molecules, which generally occur around 2800–4000 cm^−1^ [32,33]. However, these bands were not observed in Figure 6c, due to the complete evaporation of the water, explaining the existence of only the bands characteristic of CNTs.

The areas of the vibrational modes originating from ether-amine, attributed to the stretching vibrations of the C–H bonds (at 2727–2744 cm^−1^ and 2964–3082 cm^−1^), to the stretching vibrations of the N–H bonds (at 2803–2816 cm^−1^ and 3202–3306 cm^−1^) and to the NH_2_ group (at 3326 cm^−1^), are highlighted in magenta in Figure 6d,f,g. The correspondence of the sub-band located at 2535 cm^−1^ (area highlighted in dark gray) in Figure 6f was not identified. The bands located at 1425–1427 cm^−1^ in the spectra in Figure 6e,g (whose areas are highlighted in orange) may be derived from the asymmetric rocking vibrations of the H-C-H bonds associated with acetic acid (CH_3_CO_2_H) [34] present in the commercial compound Flotigam^TM^ EDA. The position, the intensity, the full width at half height (FWHM) and the integral area of the Raman bands and sub-bands obtained from the Lorentzian deconvolutions for f-CNTs (as received), BP (as produced) and BP with analytes are shown in detail in Table S2, Table S3, Table S4, and Table S5 of Supplementary Materials, respectively.

## 4. Discussion

The CNTs obtained after synthesis and those obtained after purification present Van der Waals-type intermolecular interactions, which cause their aqueous insolubility in many organic solvents. In view of this, there is a need for processes to enhance their properties and improve the dispersibility of CNTs, as is the case with functionalization which has confirmed success in increasing the stability of the dispersions produced with the start of the functionalized material [35].

In covalent functionalization, in the oxidation reaction, which uses treatment with a strong acid, such as sulfuric (H_2_SO_4_) or/and nitric (HNO_3_) acid, and an ultrasonic bath or heating, functional groups called carboxylic acids (COOH) are formed, which will covalently bond to the C atoms on the surface of the nanotubes, i.e., covalent functionalization is characterized by the bonding of functional groups to the C of the nanotubes. In this way, by including saturated C ${sp}^{3}$ atoms, the *π* bond is broken by the functionalization and the translational symmetry of the CNTs is broken, improving the interaction with polymers [36] and allowing their dispersion in an organic solvent such as isopropyl alcohol without the addition of surfactants, as illustrated in the BP production flowchart in Figure 1.

Furthermore, as demonstrated in the SEM micrographs of Figure 3, COOH functional groups improve the adhesion of CNTs to the paper framework due to their strong polar interaction (through hydrogen bonds) with the hydroxyls (OH) present in the cellulose polymer chains, resulting in a uniform film with a total thickness of around 175 µm (Figure 3a), as reported by Pinheiro et al. [37] and Ferreira et al. [18].

The response curves in Figure 4 show a periodic behavior of increase (decrease) in the variation of the relative normalized resistance in the On (Off) state, that is, during the response to analyte dripping (during sensor recovery). The BP response and recovery times for analytes EA1 and EA5 were less than 14.86 ± 1.32 min and 21.16 ± 2.35 min, respectively, when compared with EA10. The response time of a sensor is defined as the time elapsed from the moment the analyte is dripped onto the sensor until the moment when the sensor begins to be in dynamic equilibrium and reaches 90% of the saturation signal. Thus, the response time is linked to several reasons, such as the diffusion range of the adsorbate and the rate of interaction of the analyte with the adsorbent sites. Similarly, the recovery time is established by the time it takes for the sensor’s response signal to return to 90% of the initial signal variation after the analyte is removed [24,38].

The use of sensitive and selective sensors for the detection and reuse of flocculants in iron ore flotation processes can directly contribute to improving industrial efficiency and minimizing chemical reagent waste, promoting greater sustainability in the mining sector. Furthermore, proposing accurate, portable, and low-cost analytical methods, as an alternative to complex methods that depend on expensive equipment, such as chromatography, colorimetry and FTIR spectroscopy [6,7], fosters innovative technological practices that align with the goals of developing clean and environmentally friendly technologies for large-scale use, as established in the United Nations 2030 Agenda for Sustainable Development [39].

Philip et al. [40] and Fu et al. [41] demonstrated that f-CNTs exhibit greater selectivity and chemical sensitivity than pure CNTs in the detection of gas molecules. One explanation, according to Guo et al. [42], is that low-energy adsorption occurs in places with structural defects, and the complementary condensation of analyte molecules occurs. In such a way that the incorporation of an adsorbate into a defective region provides charge exchange and variation in the electrical resistance of the nanomaterial. In addition, the study showed that the resistance of a CNT is modified by three orders of magnitude after the insertion of defects, and that adsorption in these regions can favor a development in the nanomaterial’s response due to the increase in the adsorbate’s bonding energy and the charge exchange resulting from adsorption. In view of this, it can be inferred that the ability to identify the change in resistance of BP samples in the presence of different analytes was improved by the covalent functionalization process which incorporates structural defects through covalent bonding with COOH functional groups [18].

Consequently, the results of the chemiresistive test (configured as shown in Figure 2) show that the BP can distinguish between different concentrations of ether-amine in water, as well as distinguishing between deionized water and ether-amine 100 vol.%. The original pH of the samples was not changed, as the influence of pH variation with the addition of basic or acidic elements will be evaluated in future work. Due to the presence of water in the ether-amine solution, it was necessary to evaluate both separately to confirm, in part, that the BP sensor distinguishes these substances. The percentage response value increases with the ether-amine concentration; therefore, the highest value (above 700,000%) was observed for 100% ether-amine by volume, as shown in Table 1. Comparatively, Giordano et al. [43] developed a sensor based on a film of MWCNTs for measuring alcohol concentration in liquid solutions evaluating the electrical resistance of the film as a function of the concentration of isopropanol and, as a response, as the alcohol content increased, the final electrical resistance of the sensor also increased.

Additionally, the PCA results (shown in Figure 5) demonstrate the ability of the sensor element samples to recognize the analytes, based on a distinct chemoresistive response signal based on the variation in normalized electrical resistance, a fact which highlights the selective nature of the sensor element produced with BP. PCA is an important chemometric processing tool where a multidimensional data set is treated by multivariate statistics and linearly transformed into a two-dimensional (2D) or three-dimensional (3D) coordinate space, which allows the interpretation of the data by visualizing their patterns of similarities and differences [44]. On this issue, Ferreira et al. [18] had already reported the ability of the f-CNTs/cellulose BP (similar to the one developed in this research) to distinguish the intentional adulteration of Port wine with deionized water and ethyl alcohol. The response results as a function of time reported showed that the BP sensor was able to distinguish unadulterated wines from those adulterated with water and alcohol, emitting a distinct response signal depending on the type of adulterating agent added.

In Raman spectroscopy of MWCNTs, the frequency of the G band is dependent on the diameter of the tubes, increasing with decreasing diameter and decreasing with increasing diameter [45]. This way, the $G_{inner}$ sub-band is located on the right, because as it is associated with the vibrations of the innermost tubes (smaller diameter), its frequency increases compared to the $G_{outer}$ sub-band, related to the vibrations of the outermost tubes (larger diameter), which results in a reduction in its frequency, hence its location on the left. However, in the G′ band, the opposite occurs, and thus, G′_inner_ is located on the left and G′_outer_ on the right, as shown in Figure 6.

The identification of displacements in the positions of the $G_{inner}$ and $G_{outer}$ sub-bands can be indicative of the occurrence of processes suffered by the material such as compression, stretching, plastic deformation, etc. which generate changes in the C-C interatomic distances (due to the sensitivity of the G band to these changes) or related to electronic transfer (doping) [28,46]. Analysis of the position of ${G^{\prime }}_{inner}$ and ${G^{\prime }}_{outer}$ sub-bands also enable evaluation of electronic interactions or stress suffered by the material (in the latter case, the band is called “2D”).

As for the vibrational response, as shown in Table 2, the greatest variations compared to the f-CNTs (as received) occurred in the ${G^{\prime }}_{outer}$ subband, where redshifts were observed (i.e., for lower frequencies and longer wavelengths). In the case of the BP + EA1, BP + EA5, and BP + EA10 samples, these results indicate n-type charge transfers (electrons) donated by ether-amine to the most external layers of the f-MWCNT present in BP. This may have corroborated the redshift of 4 cm^−1^ in the $G_{outer}$ sub-band of the BP + EA5 sample due to electrostatic repulsion between the electrons donated by the analyte and the electronegative region of the COOH functional group of the f-CNTs, which may promote the widening of the C-C interatomic distances, causing a reduction in vibrational frequencies, shifting them to the low frequency region. According to Jório and Saito [47], changes above 2 cm^−1^ are considered significant. Consequently, only variations above this value were considered blueshifts or redshifts.

Despite the scarcity of Raman spectroscopy studies for ether-amines, the vibrational modes attributed to the stretch vibrations of the C-H bonds in Figure 6d,f,g are consistent with the vibration patterns for methylamine, diethylamine, and trimethylamine presented by Hata [48]. Similarly, the bands attributed to stretching vibrations of N-H bonds and the NH_2_ group agree with the spectra obtained by Puranik and Ramiah [49] for toluidines and amino-acetophenone. Modes attributed to the presence of acetic acid are expected, since it is used to neutralize the ether-amine, forming the ethermonoamine acetate salt, which makes this commercial compound in an aqueous medium [50]. These results are consistent with the FTIR adsorption bands for amine ether molecules, as reported by Lima, Brandrão, and Peres [34], Gouvêa Júnior et al. [51], and Budemberg [52].

**Figure 7:**
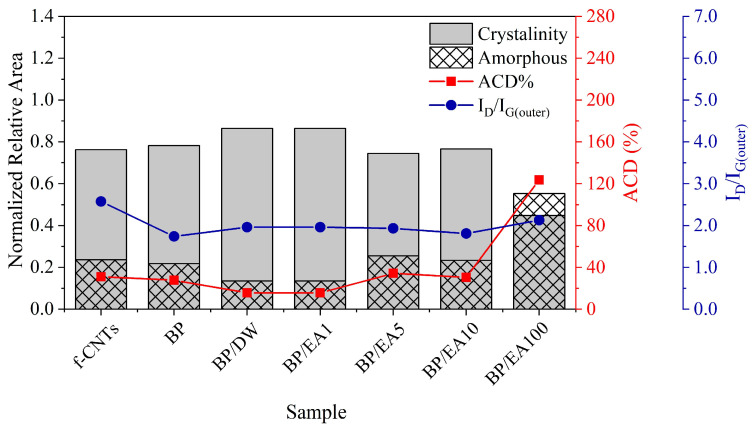
Intensity ratios of the sub-bands related to defects and graphitization of the outermost tubes (blue circles), degree of amorphous carbon (red squares), and normalized relative areas corresponding to the amorphous and crystallinity of the sample.

The ratios between the intensities of the sub-bands relative to the D and G bands, i.e., ${ID/IG}_{outer}$ and ${ID/IG}_{inner}$, which are indicative of the structural crystallinity of the CNTs and the concentration of internal and external defects, are shown in Table S6 of Supplementary Materials. Additionally, Figure 7 shows that 100 vol.% ether-amine (EA100) induces a loss of crystallinity in the BP, having a reduction from approximately 59% and 42% in the normalized relative area of crystallinity when compared to f-CNTs (as received) and BP (as produced), respectively, indicating a decrease in ${sp}^{2}$ forms and an increase in ${sp}^{3}$ forms. Thus, although ether-amine acts as an electron donor for MWCNTs, it introduces amorphous carbons that block the conduction channels and, consequently, reduce the electrical conductivity of BP instead of increasing it.

Corroborating this, Figure 7 shows an increase of about 129% and 150% in the normalized relative area of the D_M_ sub-band (amorphous carbon area, filled with hatches) for BP in the presence of 100 vol.% ether-amine compared to f-CNTs (as received) and BP (as produced), respectively. In addition, the amorphous carbon degree (ACD) percentage results for BP + EA100 were 123%, indicating increased amorphous carbon content in the sample induced by the large amount of ether-amine. ACD% makes it possible to assess the degree of amorphous carbon in the samples by means of the ratio between the integral area of the D_M_ sub-band (referring to ${sp}^{3}$ sites) and the sum of the integral areas of the $G_{inner}$ and $G_{outer}$ sub-bands (referring to ${sp}^{2}$ sites) [29].

## 5. Conclusions

The development of the sensor element based on f-CNTs for the detection of ether-amine 100 vol.% and with different concentrations, such as 1.0 vol.%, 5.0 vol.% and 10 vol.%; and deionized water, proved to be accessible and efficient, as well as flexible and responsible, so that the production, handling and relevant results do not require complicated and expensive techniques [53,54]. The nanostructured film sensor obtained by covalently functionalizing the MWCNTs with COOH showed good dispersion and adhesion to the cellulose fibers of the filter paper, as evidenced by scanning electron microscopy.

Despite the difficulty and lack of vibrational analysis studies related to ether- amine, it was possible to obtain significant results by Raman spectroscopy, through the interaction between the ether- amine molecules and the CNTs from BP, where the vibrational modes at 2727–2744 cm^−1^ and 2964–3082 cm^−1^ can be imposed on the stretching vibrations of the C-H bonds, the bands at 2803–2816 cm^−1^ and 3202–3306 cm^−1^ are associated with the stretching vibrations of the N-H bonds and the mode at 3326 cm^−1^ to the NH_2_ group. The bands located at 1425–1427 cm^−1^ come from the asymmetric balance vibrations of the H-C-H bonds associated with the acetic acid (CH_3_CO_2_H) present in Flotigam^TM^ EDA and in the case of the BP + EA1, BP + EA5 and BP + EA10 samples, these responses demonstrate n-type charge transfers (electrons) donated by ether-amine to the outermost layers of the f-CNTs present in BP.

The conclusions reached in the chemoresistive tests of the sensor element in the presence of different concentrations of the analyte and deionized water at room temperature showed, by means of normalized relative resistance curves, that it was possible to develop responses, response times, and recovery times. Thus, it can be inferred that the variation in the constant electrical behavior of increase/decrease is linked to the concentration of ether-amine present in each sample, so the higher the concentration, the higher the electrical resistance curve. Therefore, the results show that the sensor element is a chemoresistive material since it exhibits selective and reproducible results.

## Figures and Tables

**Table 1:** Response, response time, and recovery time of BP in the presence of each analyte.

Analyte	Response Time (min)	Recovery Time (min)	Response (%)
DW	7.76 ± 4.60	>16.58 ± 15.99	52.99 ± 2.64
EA100	43.57	-	723,974.00
EA1	11.49 ± 4.97	9.19 ± 7.89	446.25 ± 150.14
EA5	3.62 ± 0.99	8.52 ± 3.60	39,699.14 ± 9154.10
EA10	14.86 ± 1.32	21.16 ± 2.35	174,241.57 ± 130,005.32

**Table 2:** Variation in the $G_{outer},\;G_{inner},\;{G^{\prime }}_{outer},\;and\;{G^{\prime }}_{inner}$ sub-bands for BP without and with analytes compared to f-CNTs.

Reference		Variation (cm^−1^)					
Sub-Band	F-CNTs (Position)	BP	BP + DW	BP + EA1	BP + EA5	BP + EA 10	BP + EA100
G_outer_	1575	−1	−4	+1	−4	−1	−1
G_inner_	1590	+8	−3	−1	−3	+5	+13
G′_inner_	2633	−16	+2	-	−5	-	−13
G′_outer_	2669	−21	−21	−27	−27	−26	-

## Data Availability

Data are contained within the article and Supplementary Materials.

## Supplementary Materials

The following supporting information can be downloaded at: https://www.mdpi.com/article/10.3390/nano15151197/s1, Table S1: Data extracted via covariance matrix showing the eigenvalues and the variance explained by each PC; Table S2: Position data (cm^−1^) for the Raman modes obtained from Lorentzian deconvolutions; Table S3: Intensity data (10^3^ cm^−1^) for the Raman modes obtained from Lorentzian deconvolutions; Table S4: FWHM data (cm^−1^) for the Raman modes obtained from Lorentzian deconvolutions; Table S5: Integral area data (10^3^ a.u.) for the Raman modes obtained from Lorentzian deconvolutions; Table S6: Ratio between the relative intensities of the D, $G_{outer}$, $G_{inner}$, and D’ bands obtained for f-CNTs and BP without and with analytes.

## Abbreviations

The following abbreviations are used in this manuscript:

CNTs	carbon nanotubes
SWCNT	single-walled carbon nanotubes
DWCNT	double-walled carbon nanotubes
MWCNTs	multi-walled carbon nanotubes
BP	buckypaper
f-MWCNTs	functionalized multi-walled carbon nanotubes
EA	ether-amine
f-CNTs	functionalized carbon nanotubes
SEM	scanning electron microscopy
SE	secondary electron
DW	deionized water
ACD	amorphous carbon degree
