# Supplementary material for: Vibrational and Resistance Responses for Ether-Amine Solutions of the Buckypaper-Based Chemiresistor Sensor
Source: Nanomaterials (Basel). 2025 Aug 5;15(15):1197. doi: 10.3390/nano15151197 (PMC12348280; doi:10.3390/nano15151197)
Supplement: Supplementary file 1 [file nanomaterials-15-01197-s001.zip › nanomaterials-3681412-supplementary.pdf]

Supplementary Material

Table S1. Data extracted via covariance matrix showing the eigenvalues and the variance explained by each PC.

Principal Component	Eigenvalue	Variance (%)
1	2.14289	71.43
2	0.611932	20.398
3	0.24518	8.1727

Table S2. Position data (cm^{-1}) for the Raman modes obtained from Lorentzian deconvolutions.

	f-CNTs	BP	BP + DW	BP + EA1	BP + EA5	BP + EA10	BP + EA100
D″	1120 ± 3.62	1114 ± 3.18	1113 ± 1.16	1086 ± 4.55	1128 ± 2.58	1134 ± 8.13	1116 ± 0.92
D_{LA}	1289 ± 5.30	1265 ± 6.74	1283 ± 6.79	-	1271 ± 4.94	1308 ± 22.95	1210 ± 0.96
D	1324 ± 0.24	1326 ± 0.10	1325 ± 0.09	1324 ± 0.11	1323 ± 0.06	1325 ± 0.28	1329 ± 0.66
D_{LO}	1366 ± 8.97	1390 ± 4.49	1388 ± 3.69	-	-	1362 ± 16.32	-
H-C-H	-	-	-	-	1425 ± 2.29	-	1428 ± 1.62
D_{M}	1499 ± 2.56	1494 ± 4.53	1485 ± 1.75	1508 ± 6.07	1509 ± 2.05	1488 ± 6.60	1492 ± 1.27
G_{BWF}	1558 ± 2.11	1555 ± 4.83	1548 ± 2.00	1562 ± 3.42	1557 ± 1.97	1560 ± 6.97	1539 ± 0.90
G_{outer}	1575 ± 0.57	1574 ± 0.42	1571 ± 0.29	1576 ± 1.10	1571 ± 0.32	1574 ± 0.92	1574 ± 0.41
G_{inner}	1590 ± 0.97	1598 ± 0.86	1587 ± 0.60	1589 ± 1.54	1587 ± 0.91	1595 ± 1.52	1603 ± 0.33
D'	1607 ± 0.59	1610 ± 0.59	1607 ± 0.16	1608 ± 0.40	1605 ± 0.27	1607 ± 0.60	1622 ± 0.53
D+D"	2433 ± 1.91	2445 ± 2.13	2443 ± 2.74	2443 ± 1.95	2441 ± 1.81	2442 ± 1.16	-
?	-	-	-	-	-	2535 ± 0.79	-
G'_{inner}	2633 ± 2.01	2617 ± 7.25	2635 ± 3.23	-	2628 ± 4.37	-	2620 ± 4.98
G'_{outer}	2669 ± 2.63	2648 ± 1.21	2648 ± 0.79	2642 ± 0.97	2642 ± 0.20	2643 ± 0.37	-
C-H	-	-	-	2728 ± 1.83	-	2743 ± 2.78	-
N-H	-	-	-	2803 ± 2.42	-	2815 ± 4.24	2811 ± 2.92
D+G	2870 ± 4.88	2900 ± 1.07	2903 ± 0.70	2905 ± 2.52	2892 ± 0.76	2905 ± 3.50	-
C-H	-	-	-	-	-	-	2965 ± 1.92
O-H	-	-	-	3000 ± 3.38	-	-	-
C-H	-	-	-	-	-	3033 ± 6.11	-
C-H	-	-	-	-	-	-	3081 ± 0.65
O-H	-	-	-	3126 ± 1.70	-	-	-
N-H	-	-	-	-	-	-	3202 ± 1.06
O-H	-	-	-	3239 ± 0.93	-	-	-
N-H	-	-	-	-	-	-	3306 ± 1.57
NH_2	-	-	-	3326 ± 1.74	-	-	-
O-H	-	-	-	3421 ± 1.63	-	-	-

Table S3. Intensity data (10^3 cm^{-1}) for the Raman modes obtained from Lorentzian deconvolutions.

	**f-CNTs**	**BP**	**BP + DW**	**BP + EA1**	**BP + EA5**	**BP + EA10**	**BP + EA100**
D″	0.52 ± 0.03	1.00 ± 0.08	0.70 ± 0.02	0.32 ± 0.01	0.73 ± 0.04	0.39 ± 0.03	0.48 ± 0.02
D_{LA}	1.36 ± 0.28	0.58 ± 0.07	0.48 ± 0.08	-	1.84 ± 0.13	1.52 ± 1.32	1.12 ± 0.04
D	13.23 ± 0.38	16.47 ± 0.16	13.17 ± 0.11	5.27 ± 0.02	27.61 ± 0.19	9.84 ± 1.26	2.82 ± 0.07
D_{LO}	0.52 ± 0.16	0.85 ± 0.10	0.34 ± 0.03	-	-	0.54 ± 0.52	-
H-C-H	-	-	-	-	2.13 ± 0.11	-	1.54 ± 0.12
D_M	0.80 ± 0.05	0.98 ± 0.12	0.57 ± 0.03	0.26 ± 0.03	2.18 ± 0.20	0.54 ± 0.08	1.16 ± 0.14
G_{BWF}	3.11 ± 0.65	1.54 ± 0.72	1.17 ± 0.17	1.31 ± 0.64	6.31 ± 1.56	2.25 ± 1.66	1.04 ± 0.12
G_{outer}	5.14 ± 0.87	9.46 ± 0.76	6.71 ± 0.38	2.02 ± 0.82	14.27 ± 2.18	5.44 ± 1.85	1.32 ± 0.08
G_{inner}	2.07 ± 0.57	2.90 ± 0.53	2.54 ± 0.33	0.97 ± 0.47	5.01 ± 1.19	1.45 ± 0.66	0.89 ± 0.04
D'	4.12 ± 0.23	5.01 ± 0.49	5.09 ± 0.10	2.03 ± 0.07	11.39 ± 0.43	4.31 ± 0.52	0.35 ± 0.03
D+D"	0.50 ± 0.04	0.28 ± 0.05	0.36 ± 0.01	0.32 ± 0.02	0.92 ± 0.04	1.91 ± 0.07	-
?	-	-	-	-	-	3.37 ± 0.08	-
G'_{inner}	1.95 ± 0.11	0.44 ± 0.25	1.43 ± 0.37	-	1.72 ± 0.37	-	0.39 ± 0.02
G'_{outer}	0.66 ± 0.14	4.41 ± 0.23	2.05 ± 0.37	3.52 ± 0.07	8.96 ± 0.38	7.40 ± 0.11	-
C-H	-	-	-	2.03 ± 0.24	-	2.36 ± 0.35	-
N-H	-	-	-	2.21 ± 0.29	-	1.68 ± 0.51	0.77 ± 0.05
D+G	0.48 ± 0.02	1.18 ± 0.02	1.06 ± 0.01	2.29 ± 0.37	1.84 ± 0.02	3.07 ± 0.33	-
C-H	-	-	-	-	-	-	1.61 ± 0.10
O-H	-	-	-	2.25 ± 0.37	-	-	-
C-H	-	-	-	-	-	1.02 ± 0.12	-
C-H	-	-	-	-	-	-	3.43 ± 0.12
O-H	-	-	-	3.62 ± 0.28	-	-	-
N-H	-	-	-	-	-	-	1.69 ± 0.07
O-H	-	-	-	4.91 ± 0.27	-	-	-
N-H	-	-	-	-	-	-	0.68 ± 0.04
NH_2	-	-	-	2.07 ± 0.17	-	-	-
O-H	-	-	-	0.90 ± 0.05	-	-	-

Table S4. FWHM data (cm^{-1}) for the Raman modes obtained from Lorentzian deconvolutions.

	**f-CNTs**	**BP**	**BP + DW**	**BP + EA1**	**BP + EA5**	**BP + EA10**	**BP + EA100**
D″	115.70 ± 6.41	157.41 ± 9.39	95.92 ± 2.04	200.76 ± 8.98	102.24 ± 4.91	184.84 ± 12.86	77.92 ± 2.02
D_{LA}	71.17 ± 3.98	100.42 ± 12.47	84.89 ± 5.87	- -	130.87 ± 6.44	95.92 ± 6.13	139.17 ± 3.16
D	47.30 ± 0.70	48.16 ± 0.30	48.45 ± 0.23	52.49 ± 0.19	49.94 ± 0.17	43.34 ± 1.24	148.88 ± 1.97
D_{LO}	67.97 ± 9.40	104.01 ± 13.30	76.58 ± 8.01	- -	-	89.00 ± 18.15	-
H-C-H	-	-	-	- -	121.60 ± 4.57	-	118.84 ± 4.09
D_M	64.06 ± 4.47	101.78 ± 11.52	65.98 ± 3.98	78.72 ± 7.12	78.40 ± 4.89	111.80 ± 14.65	80.92 ± 4.51
G_{BWF}	33.93 ± 1.90	39.00 ± 5.47	39.14 ± 2.48	29.64 ± 2.69	35.05 ± 1.72	43.24 ± 3.41	56.12 ± 3.20
G_{outer}	24.12 ± 2.09	30.59 ± 1.07	26.66 ± 0.75	23.09 ± 4.17	25.47 ± 1.32	29.43 ± 2.77	39.34 ± 1.40
G_{inner}	18.29 ± 2.70	19.88 ± 1.97	22.61 ± 1.41	17.43 ± 2.97	24.74 ± 2.35	20.99 ± 4.74	24.93 ± 1.01
D'	24.75 ± 0.52	21.22 ± 0.49	21.29 ± 0.21	22.03 ± 0.50	27.00 ± 0.25	20.85 ± 0.56	16.43 ± 0.91
D+D"	50.45 ± 2.90	23.03 ± 3.21	139.18 ± 4.60	69.47 ± 3.39	116.20 ± 3.63	99.01 ± 1.77	-
?	-	-	-	-	-	123.15 ± 2.55	-
G'_{inner}	68.39 ± 1.70	50.23 ± 8.60	116.71 ± 4.94	-	187.65 ± 12.31	-	302.79 ± 4.79
G'_{outer}	40.57 ± 4.48	66.32 ± 0.79	57.35 ± 2.51	131.23 ± 0.98	70.43 ± 0.92	112.02 ± 1.13	-
C-H	-	-	-	105.20 ± 4.85	-	111.13 ± 6.08	-
N-H	-	-	-	133.47 ± 8.29	-	125.70 ± 15.38	233.04 ± 7.11
D+G	242.53 ± 8.73	109.94 ± 1.97	129.16 ± 1.18	145.46 ± 9.34	137.67 ± 1.37	162.76 ± 7.25	-
C-H	-	-	-	-	-	-	206.10 ± 5.29
O-H	-	-	-	164.96 ± 10.67	-	-	-
C-H	-	-	-	-	-	196.93 ± 5.48	-
C-H	-	-	-	-	-	-	181.08 ± 2.37
O-H	-	-	-	181.15 ± 6.50	-	-	-
N-H	-	-	-	-	-	-	178.81 ± 3.59
O-H	-	-	-	145.03 ± 3.56	-	-	-
N-H	-	-	-	-	-	-	133.82 ± 2.67
NH_2	-	-	-	119.74 ± 4.31	-	-	-
O-H	-	-	-	94.20 ± 2.52	-	-	-

Table S5. Integral area data (10^3 a.u.) for the Raman modes obtained from Lorentzian deconvolutions.

	**f-CNTs**	**BP**	**BP + DW**	**BP + EA1**	**BP + EA5**	**BP + EA10**	**BP + EA100**
D″	79.82	197.58	91.49	70.88	102.36	89.89	52.75
D_{LA}	145.68	85.91	60.13	-	346.12	215.79	217.12
D	957.20	1212.39	975.09	421.66	2105.45	653.70	605.28
D_{LO}	53.83	131.55	39.51	-	-	71.50	-
H-C-H	-	-	-	-	385.02	-	273.17
D_M	78.38	150.20	57.52	31.13	260.40	90.09	142.35
G_{BWF}	163.95	92.93	70.98	60.35	343.04	150.59	89.68
G_{outer}	193.07	449.57	278.52	72.72	565.72	248.63	80.62
G_{inner}	59.11	90.06	89.58	26.42	192.99	47.36	34.54
D'	158.83	165.72	169.00	69.83	478.55	140.02	8.97
D+D"	39.29	10.15	75.52	34.24	163.78	289.47	-
?	-	-	-	-	-	630.92	-
G'_{inner}	205.57	34.61	254.58	-	482.47	-	171.30
G'_{outer}	41.42	451.34	181.76	701.10	972.48	1263.90	-
C-H	-	-	-	325.17	-	399.50	-
N-H	-	-	-	445.15	-	319.94	264.04
D+G	168.47	197.28	205.45	499.35	380.72	745.74	-
C-H	-	-	-	-	-	-	486.37
O-H	-	-	-	550.76	-	-	-
C-H	-	-	-	-	-	294.61	-
C-H	-	-	-	-	-	-	908.53
O-H	-	-	-	954.78	-	-	-
N-H	-	-	-	-	-	-	436.25
O-H	-	-	-	1036.75	-	-	-
N-H	-	-	-	-	-	-	131.56
NH_2	-	-	-	359.45	-	-	-
O-H	-	-	-	121.38	-	-	-

Table S6. Ratio between the relative intensities of the D, G_{outer}, G_{inner} and D′ bands obtained for f-CNTs and BP without and with analytes.

	**f-CNTs**	**BP**	**BP + DW**	**BP + EA1**	**BP + EA5**	**BP + EA10**	**BP + EA100**
ID/IG(outer)	2.57	1.74	1.96	2.61	1.94	1.81	2.13
ID/IG(inner)	6.39	5.67	5.18	5.43	5.51	6.80	3.17
ID/ID′	3.21	3.29	2.59	2.59	2.42	2.28	8.06
